# Low Dose of Genistein Alleviates Mono-(2-Ethylhexyl) Phthalate-Induced Fetal Testis Disorder Based on Organ Culture Model

**DOI:** 10.1155/2020/4569268

**Published:** 2020-05-27

**Authors:** Tong-Dian Zhang, Yu-Bo Ma, He-Cheng Li, Tie Chong, Zi-Ming Wang, Lian-Dong Zhang

**Affiliations:** Department of Urology, The Second Affiliated Hospital of Xi'an Jiaotong University, Xi'an, Shaanxi 710004, China

## Abstract

Mono-(2-ethylhexyl) phthalate (MEHP) and genistein have been classified as endocrine-disrupting chemicals (EDCs) which interfere with the differentiation and development of the male reproductive system. However, how these two EDCs would affect fetal rat testis development at a low dose was rarely studied. In this study, we established the organ culture system and applied it to evaluate testicular effects following multiple EDC exposure at a low dose. 15.5 days postcoitum fetal rat testes were dissected, cultured, and exposed to vehicle (control), GEN (1 *μ*mol/L, G), MEHP (1 *μ*mol/L, M), or GEN (1 *μ*mol/L)+MEHP (1 *μ*mol/L, G+M). Testicular cell markers, testosterone concentration, redox state, testicular histology, and testicular ultrastructure were evaluated. Our results showed that a low dose of MEHP suppressed the development of Sertoli cells, Leydig cells, and gonocytes by triggering oxidative injuries, which was consistent with the ultrastructural findings. However, coadministration of genistein at a low dose could partially attenuate MEHP-induced fetal testis damage through antioxidative action. Cotreatment of genistein at a low dose may have a promising future on its protecting role for attenuating other EDC-induced reproductive disorders during early life. Based on the results, it can be speculated that dietary intake of isoflavones may make the fetal testis less susceptible to phthalate-induced injury.

## 1. Introduction

In the last two decades, there have been growing concerns on endocrine-disrupting chemical- (EDC-) related male reproductive system disorder. Multiple evidences suggested that EDCs can interfere with the production, release, transport, metabolism, binding action, or elimination of the natural hormones in the body, exhibiting potentially deleterious effects on the development of the male reproductive system [1]. Although extensive studies have been done to make clear the reproductive effects of single EDC exposure, however, knowledge gap still exists on testicular effects following multiple EDC exposure at low doses.

Several common EDCs, including derivatives of paints, plastics, and natural soy-derived phytoestrogens, have been detected in amniotic fluid, cord blood, and breast milk [2–4], indicating that humans and animals are exposed to a myriad of potentially harmful substances in early life. However, the combined effects after exposure to at least two kinds of EDCs have rarely been investigated [5]. Genistein (GEN), a kind of soy isoflavones commonly present in the Asian diet [6], and mono-(2-ethylhexyl) phthalate (MEHP), the metabolite of the widely used plasticizer di(2-ethylhexyl) phthalate (DEHP), are two kinds of the most prevalent EDCs which act via different mechanisms [7]. In previous studies, genistein was categorized as a weak estrogenic phytoestrogen, but more recently, it was found to be a potential antioxidant [8]. It is notable that genistein could exert biphasic effects on male fertility by promoting acrosome reaction at lower doses while suppressing acrosome reaction at higher doses [9]. Soy isoflavones including genistein were confirmed to enhance cellular antioxidative system and alleviate the oxidative injury induced by EDCs like cadmium, TPA [6, 10, 11]. DEHP and MEHP were reported to suppress fetal testosterone biosynthesis by activating peroxisome proliferator-activated receptors (PPARs) [12]. Zhou et al. [13] found that the reactive oxygen species (ROS) level increased after MEHP exposure for one hour in MA-10 Leydig cells and hypothesized that impairment of mitochondrial function may contribute to the oxidative stress. Epidemiological study also revealed that urinary malondialdehyde (MDA) concentration was significantly correlated with DEHP metabolite levels in prepubertal children [14].

Fetal period is a critical stage of the male reproductive system development; in this stage, germ cell and testicular somatic cells differentiate and proliferate, finally leading to the formation of mature spermatozoa during puberty. During fetal testis development, fetal Leydig cells begin to produce testosterone around 15.5 days postcoitum (dpc) in the rat and testosterone production decreases from 18.5 dpc onwards [15]. It was identified that the critical EDC exposure window for reproductive tract lesion encompasses approximately 16~18 dpc [16]. Moreover, several fetal characteristics including minimal detoxifying capacity and lack of blood-testis barrier make the developing male gonads particularly sensitive to EDCs capable of crossing the placenta [2]. EDC exposure during this stage would account for changes in the total activity of the involved hormones, resulting in long-term adverse consequences that may be apparent during puberty or even adult life [17, 18].

Previous studies mainly focused on the reproductive outcomes after single EDC exposure in vitro, ex vivo, and in vivo [19–21]; however, how multiple EDCs at low doses would disrupt fetal testis development was scarcely investigated. Furthermore, the great majority of in vivo studies were conducted in utero and cannot distinguish direct and indirect effects of EDCs. Our in vivo study demonstrated that genistein could normalize DEHP-induced neonatal effects at doses equivalent to human exposure level [22], highlighting the importance of assessing reproductive impacts of EDC exposure at low doses. However, because of the difficulty to obtain fetal testis and lack of stable culture system, fetal testis exposure risk evaluation based on ex vivo model was rarely reported. Our previous study showed that somatic cells and germ cells of 15.5 dpc fetal rat testis ex vivo can develop in the pattern similar to that observed in vivo, in a modified agarose culture system in which KSR served as a supplement of a culture medium [23]. The system is easy to manipulate and could better mimic the microenvironment of the in situ testis, which makes it possible to investigate the combined effects following a low dose of genistein and MEHP exposure. Considering oxidative stress is a common pathological process involved in EDC-induced testicular cell injury [24, 25], we hypothesized that a low dose of genistein exposure would exert its antioxidative role in ex vivo fetal testis, which may contribute to the alleviation of toxic effect induced by MEHP. Parameters including testicular histology, testosterone production, and ultrastructure of cultured fetal testes were examined; gene expression of testicular cell markers and antioxidative defense system was also evaluated, hoping to gain insight into the early molecular events involved in a low dose effect.

## 2. Materials and Methods

### 2.1. Animal Feeding and Sample Collection

Prior to initiation of the study, the experimental protocol was reviewed and approved by the Committee on Animal Research and Ethics of Xi'an Jiaotong University (Xi'an, China). Two-month-old specific pathogen-free (SPF) Sprague-Dawley rats obtained from the Experimental Animal Center of Xi'an Jiaotong University were kept under a 12 h light/dark cycle with ad libitum access to purified water and soy-free diet. Vaginal smears were conducted to judge female rat estrous cycle. Female rats which were in proestrus and estrus were caged with the male for the night in the proportion of 2 : 1. Vaginal smears were collected in the morning daily, and the day of sperm detection was considered as 0.5 dpc.

On 15.5 dpc, pregnant female Sprague-Dawley rats were anesthetized by intraperitoneal injection of 2% (*w*/*v*) sodium pentobarbital at the dose of 40 mg/kg body weight (Sigma-Aldrich Inc., St. Louis, USA). The fetuses were removed from the uterus and placed on ice quickly. For fetuses older than 13.5 dpc (rat) or 11.5 dpc (mouse), the testes could be distinguished morphologically from the ovaries as fetal testes were rounder and contained testicular vessels [26]. The fetal testes were isolated aseptically from the 15.5 dpc rats under an anatomical microscope and then immediately transferred into a culture medium on ice.

### 2.2. Organ Culture Protocol and Treatment

As previously described [27], agarose gel was prepared before the initiation of organ culture. Agarose powder was dissolved in double-distilled water to 1.5% (*w*/*v*) and autoclaved. Hot agarose solution was poured into dishes to form a 5 mm thick agarose gel. The gel was then cut into 10 × 5 × 5 mm^3^ pieces using a sterile blade and transferred into a culture medium 12 h before use.

The isolated fetal testes were transferred to the surface of agarose gel which was placed on Millicell insert (PICM-01250, Millipore Corp., USA) half-soaked in the 24-well plate in which 600 *μ*L of culture medium was added. The culture medium used was Dulbecco's Modified Eagle's Medium (DMEM) mixed 1 : 1 with Ham's F12 (DMEM/F12) medium (TransGen Biotech, Beijing, China) supplemented with 10% (*v*/*v*) KnockOut™ Serum Replacement (KSR, Gibco, USA), penicillin (100 IU/mL), and streptomycin (100 IU/mL) (TransGen Biotech, Beijing, China). Each gel stand was loaded with one testis, and a medium was collected and changed daily. The fetal testes were incubated for 4 days under the condition of 37°C in a humidified atmosphere containing 95% air and 5% CO_2_. The day starting organ culture was counted as day 0 (d0), and the following days were numbered as d1, d2, d3, and d4 subsequently.

MEHP (CAS:4376-20-9, obtained from AccuStandard Inc, Connecticut, USA) and genistein (CAS: 446-72-0, obtained from Sigma-Aldrich Inc., St. Louis, USA) were dissolved in 99.5% pure dimethylsulfoxide (DMSO, CAS: 67-68-5, obtained from Sigma-Aldrich Inc., St. Louis, USA) into stock solutions of 100 mmol/L, which were then diluted into final treatment concentration. Cultured fetal testes were exposed to vehicle (control), GEN (1 *μ*mol/L, G), MEHP (1 *μ*mol/L, M), or GEN(1 *μ*mol/L)+MEHP (1 *μ*mol/L) (G+M) separately. 600 *μ*L of culture medium was added to each well. After culture for 4 days, samples and culture medium were collected for further analysis.

Four fetuses from the same mother rats were dissected and accepted different treatment according to the design, and each group had 6 fetal rats in total. We obtained two testes from the same fetal rats, one testis was used for PCR test, and the other testis from the same fetal rat was used for H&E staining and ultrastructural study. Six samples of the culture medium from each group with 3 replicates per sample were used for testosterone test and redox state evaluation. The design could minimize differences between groups and keep better homogeneity.

### 2.3. RNA Extraction and Quantitative Real-Time PCR

Fetal testes RNA was extracted using the EasyPure RNA Kit (Transgen Biotech, China). cDNA was synthesized from isolated RNA using the RevertAid™ First Strand cDNA Synthesis Kit (Thermo Fisher Scientific, US). Quantitative real-time PCR (qPCR) was performed using the Bio-Rad Real-Time PCR System (IQ5, Bio-Rad). Gapdh was used as an endogenous control and for normalization of gene targets. The 1relative gene expression was analyzed using the 2^−*Δ*Ct^ algorithm. The genes and primer sequences are listed in Table 1.

### 2.4. Testosterone Concentration of a Culture Medium

Culture medium collected on d1, d2, d3, and d4 was stored at -80°C and measured using a testosterone radioimmunoassay kit (Tianjin Nine Tripods Medical and Bioengineering Co., Ltd., Tianjin, China) according to the manufacturer's instructions.

### 2.5. Evaluation of Medium Redox State

After fetal testes were treated for 4 days, a medium was collected for further redox state analysis. Total antioxidant capacity (T-AOC), superoxide dismutase (SOD), glutathione (GSH), malondialdehyde (MDA), and hydroxyl free radical scavenging capacity (HFRSC) were evaluated using the clinical chemistry assay kits (T-AOC, SOD, GSH, and MDA from Nanjing Jiancheng Bioengineering Institute, China; HFRSC from Suzhou Comin Biotechnology Co. Ltd.) according to the manufacturer's instructions to test cultured fetal testicular redox state.

T-AOC was determined by the ferric reducing/antioxidant power assay and detected at 520 nm using the spectrophotometer, and the final concentration was expressed as nmol/mL.

SOD activity was measured by water soluble tetrazolium salt assay (WST-1); the absorbance was scanned at 450 nm using a microplate reader. The final result was expressed as U/mL.

MDA was analyzed using the thiobarbituric acid reactive substance (TBARS) method, and the absorbance was measured with the ultraviolet spectrometer at 532 nm against blank prepared by distilled water, and result was expressed as nmol/mL.

HFRSC was tested using the hydroxyl free radical scavenging capacity assay kit, the absorbance was measured using a spectrometer at the wavelength of 536 nm against blank prepared by distilled water, and the final result was expressed as U/mL.

GSH content was measured using dithionitrobenzoic acid reagent, and the absorbance was scanned at 405 nm using a microplate reader; GSH content was calculated as T − GSH − 2 × GSSG, and the final results were expressed as *μ*mol/mL.

### 2.6. Testicular Histology

Following fixation in 4% (*w*/*v*) paraformaldehyde fixative solution for 6 h at 4°C, testes collected on d4 were transferred to ethanol and xylene, embedded in paraffin, and cut into 5 *μ*m sections. Sections were stained with 0.2% (*w*/*v*) hematoxylin for 2 min and 0.5% (*w*/*v*) eosin for 10 min and evaluated under light microscopy. Those evaluations were performed by an experienced investigator blind to treatment.

### 2.7. Ultrastructural Study of Cultured Fetal Testis

The harvested testes were promptly washed with 0.1 mol/L phosphate-buffered saline (PBS), immersed in 2.5% (*w*/*v*) phosphate-buffered glutaraldehyde and 4% (*w*/*v*) phosphate-buffered paraformaldehyde for 2 h at 4°C. Then, testes were washed with 0.1 mol/L phosphate buffer for 30 min, postfixed in 1% (*w*/*v*) phosphate-buffered osmium tetroxide for 2 h at 4°C. Samples were then embedded, sectioned, and double stained with uranyl acetate and lead hydroxide and analyzed using an H-7650 transmission electron microscope at 80 kV (Hitachi, Japan).

### 2.8. Statistical Analysis

Data were expressed as the mean ± standard deviation and analyzed using SPSS 15.0 (SPSS Inc., Chicago, IL, USA). Normality and homogeneity of variances were evaluated prior to statistical analysis. Data were analyzed by one-way analysis of variance (ANOVA), and multiple comparisons were done between different culture times by LSD when equal variances were assumed otherwise followed by Games-Howell. Differences were considered to be statistically significant at the probability level of 5% (*P* < 0.05).

## 3. Results

### 3.1. Gene Expression of Germ Cell Markers

**Figure 1:**
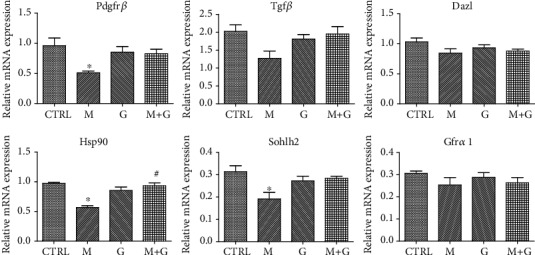
Gene expression of fetal germ cell markers. ^∗^Significantly different from CTRL at *P* < 0.05; ^#^significantly different from group M at *P* < 0.05.

Gene expression of germ cell markers is shown in Figure 1. No significant changes of Tgf*β*, Gfr*α*1, and Dazl were observed between each group (*P* > 0.05). Exposure to MEHP caused a significant decrease in Sohlh2, Hsp90, and Pdgfr*β* expression compared with control (*P* < 0.05), while the combined exposure of MEHP and genistein showed an increase in Hsp90 expression compared to MEHP single exposure (*P* < 0.05), which manifests that genistein may exert its protective role in fetal germ cell development.

### 3.2. Gene Expression of Sertoli Cell Markers

**Figure 2:**
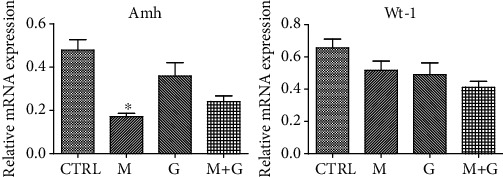
Gene expression of Sertoli cell markers. ^∗^Significantly different from CTRL at *P* < 0.05; ^#^significantly different from group M at *P* < 0.05.

The expression of Sertoli cell markers of each group is shown in Figure 2. Wt-1 expression showed no significant difference between each group (*P* > 0.05). Amh was downregulated after MEHP exposure for 4 days compared with the control group (*P* < 0.05) while the combined exposure to MEHP and genistein showed no significant difference with the control group (*P* > 0.05).

### 3.3. Gene Expression of Leydig Cell Markers

**Figure 3:**
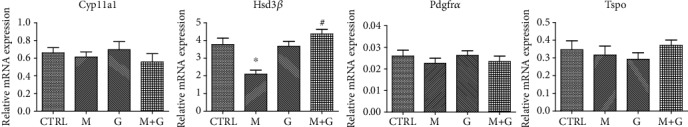
Gene expression of Leydig cell markers. ^∗^Significantly different from CTRL at *P* < 0.05; ^#^significantly different from group M at *P* < 0.05.

The gene expression of Leydig cell markers after cultured for 4 days is shown in Figure 3. The expression of Cyp11a1, Tspo, and Pdgfr*α* showed no significant difference between each group (*P* > 0.05). Compared to the control group, MEHP treatment downregulated the expression of Hsd3*β* (*P* < 0.05) while the combination of genistein and MEHP exhibited a significant increase compared with MEHP exposure (*P* < 0.05), indicating that genistein may alleviate fetal Leydig cell injury induced by MEHP.

### 3.4. Gene Expression of Nrf2 and Antioxidative Genes

**Figure 4:**
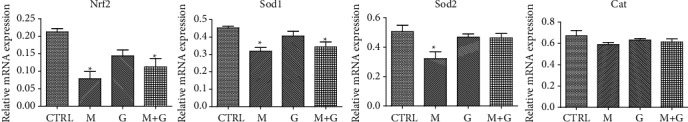
Gene expression of Nrf2 and downstream antioxidative genes. ^∗^Significantly different from CTRL at *P* < 0.05; ^#^significantly different from group M at *P* < 0.05.

The expression of Nrf2 and downstream antioxidative genes is shown in Figure 4. MEHP exposure and the combined exposure downregulated Nrf2 and Sod1 expression (*P* < 0.05). Sod2 expression in the MEHP-treated group was significantly lower than that of control (*P* < 0.05). No significant alternation of Cat was found in each treated group compared with control (*P* > 0.05).

### 3.5. Testosterone Concentration of Cultured Fetal Testes

**Figure 5:**
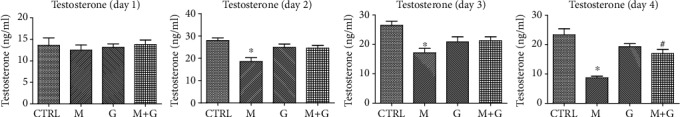
Testosterone concentration of cultured fetal testes. ^∗^Significantly different from CTRL at *P* < 0.05; ^#^significantly different from group M at *P* < 0.05.

Testosterone secreted by cultured fetal testes on days 1, 2, 3, and 4 was separately measured (Figure 5). On day 1, no significant difference was found between the four groups (*P* > 0.05). In the subsequent 3 days, it was found that MEHP significantly decreased the testosterone production compared with control (*P* < 0.05). Although still lower than control, coexposure to MEHP and genistein significantly elevated testosterone concentration compared with MEHP single exposure on day 4 (*P* < 0.05).

### 3.6. Analysis of Medium Redox State

**Figure 6:**
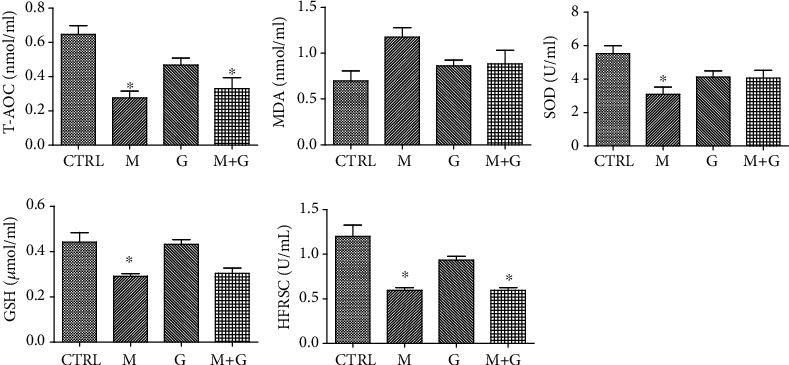
Effects of GEN and MEHP exposure on medium redox state. ^∗^Significantly different from CTRL at *P* < 0.05; ^#^significantly different from group M at *P* < 0.05.

The medium redox state in each group is shown in Figure 6. MEHP treatment resulted in significant reduction of T-AOC, SOD activity, GSH, and HFRSC compared with control (*P* < 0.05). The combination of genistein and MEHP also exhibited a significant decrease of T-AOC and HFRSC compared with control (*P* < 0.05), which indicates that although genistein could partially alleviate the oxidative injury, it cannot completely recover fetal testicular redox balance.

### 3.7. Testicular Histology

**Figure 7:**
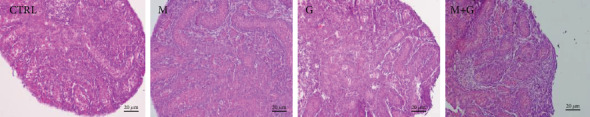
H&E staining of fetal testis cultured for 4 days. H&E staining showed an intact testicular structure and normal appearance of seminiferous tubules; no apparent necrosis or vacuole was visible in all groups; formation of tubular lumens was still not observed. 200x magnification. Scale bars indicate 20 *μ*m.

Testicular sections on d4 are shown in Figure 7. After cultured for 4 days, H&E staining of fetal testis showed an intact testicular structure and normal appearance of seminiferous tubules; no apparent necrosis or vacuole was visible in all groups. Among all cell types, gonocytes were located in the center and Sertoli cells were located in the periphery of the tubules, and formation of tubular lumens was still not observed in the four groups, manifesting that fetal testis histology was not apparently disrupted after exposure to a low dose of EDCs.

### 3.8. Ultrastructural Study of Gonocytes on d4

**Figure 8:**
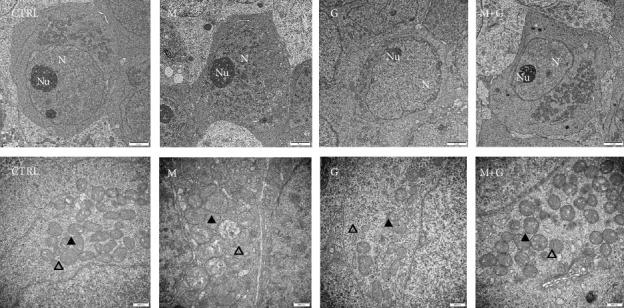
Ultrastructural study of gonocytes on d4. Control, group G, and group G+M exhibited normal gonocyte characteristics; endoplasmic reticulum and mitochondria were well defined. MEHP exposure induced apparent mitochondrial swelling and disappearance of the mitochondrial cristae. N: nuclei; Nu: nucleolus; ▲: mitochondria; △: endoplasmic reticulum. The upper row: 10000x, the lower row: 30000x magnification; scale bars indicate 2 *μ*m and 500 nm, respectively.

The ultrastructure of gonocytes is shown in Figure 8. In groups G and G+M, gonocytes were round to oval in shape and still far away from the basement membrane. The nuclei were round and located in the center. Further observation showed endoplasmic reticulum, and mitochondria were well defined and distributed throughout the cytoplasm. MEHP exposure induced apparent mitochondrial swelling and part of the mitochondrial cristae disappeared inside the gonocytes, and chromatin condensation was also observed in the region around the nucleolus.

### 3.9. Ultrastructural Study of Sertoli Cells on d4

**Figure 9:**
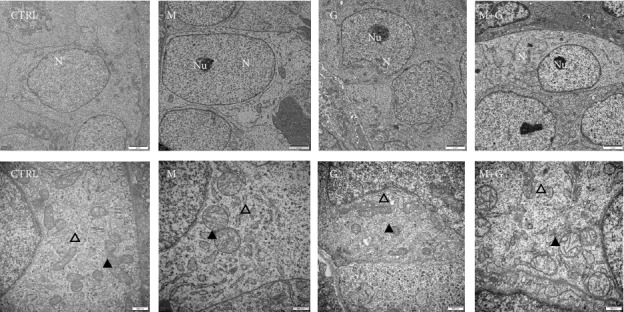
Ultrastructural study of Sertoli cells on d4. Sertoli cells in control and group G showed normal ultrastructure. In contrast, Sertoli cells in groups M and G+M exhibited apparent mitochondrial swelling and part of the mitochondrial cristae disappeared. N: nuclei; Nu: nucleolus; ▲: mitochondria; △: endoplasmic reticulum. The upper row: 10000x, the lower row: 30000x magnification, scale bars indicate 2 *μ*m and 500 nm, respectively.

The ultrastructure of Sertoli cells located in the periphery of seminiferous tubules is shown in Figure 9. Sertoli cells in control and group G were round to oval in shape and adjacent to the basement membrane; no obvious swelling in mitochondria and endoplasmic reticulums was found. In contrast, Sertoli cells in groups M and G+M exhibited apparent mitochondrial swelling and part of the mitochondrial cristae disappeared, manifesting that a low dose of MEHP might disturb Sertoli cell mitochondrial function and genistein cannot completely alleviate MEHP-induced mitochondrial injury in Sertoli cells.

### 3.10. Ultrastructural Study of Leydig Cells and Basement Membrane on d4

**Figure 10:**
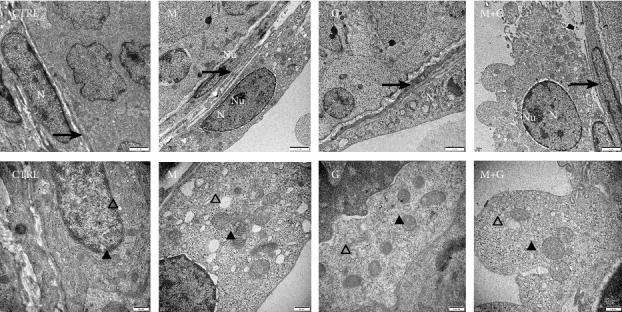
Ultrastructural study of Leydig cells and basement membrane on d4. Normal ultrastructure of Leydig cells and intact basement membrane were observed in control, group G, and group G+M, and also, no obvious swelling in mitochondria and endoplasmic reticulums was observed. By contrast, MEHP exposure induced mitochondrial edema and dilation of smooth endoplasmic reticulum. N: nuclei; Nu: nucleolus; ▲: mitochondria; △: endoplasmic reticulum, →: basement membrane. The upper row: 10000x, the lower row: 30000x magnification, scale bars indicate 2 *μ*m and 500 nm, respectively.

The ultrastructure of Leydig cells and basement membrane is shown in Figure 10. Normal ultrastructure of Leydig cells and intact basement membrane were observed in control, group G, and group G+M; also, no obvious swelling in mitochondria and endoplasmic reticulums was observed. By contrast, MEHP exposure induced mitochondrial edema and dilation of smooth endoplasmic reticulum, manifesting that single exposure to a low dose of MEHP exhibited obvious alternation in ultrastructure of Leydig cells which was absent in the combined exposure group.

## 4. Discussion

Fetal period has classically been defined as a sensitive window for male reproductive system development. The fetal testis development from a sexually undifferentiated genital ridge to the initiation of spermatogenesis involves a series of events that may affect all types of testicular cells [28]. It is widely accepted that testicular dysgenesis syndrome (TDS), which includes decreasing semen quantity, increasing incidence of cryptorchidism and hypospadias, arises from impaired development and reprogramming of testis progenitor cells during fetal window. Among all factors, fetal exposure to EDCs was thought to contribute to decline in male reproductive potential and increased incidence of developmental abnormalities due to impaired testosterone production and Sertoli cell function [29, 30].

Although extensive researches have been done to make clear the toxic effects of single chemical exposures, few studies have examined the reproductive effects after EDC coexposure, especially at low doses [18]. Although in vitro approaches have been used for analyzing the effects of EDCs on rat fetal testis, obvious limitations including poor survival and inability to differentiate still exist [31]. In contrast, the organ culture system could preserve testicular architecture, and thus, intercellular communications as well as fetal testis development could be better maintained. However, the difficulty to obtain fetal testis and a stable organ culture system makes the ex vivo studies have been lacking.

In the present study, we used a modified agarose organ culture system which could support normal differentiation of the fetal gonad for at least 4 days. During the ex vivo culture, testicular structure and development were well maintained. Differentiation of Sertoli and Leydig cells was precisely regulated by critical genes to ensure production of anti-Müllerian hormone (AMH) and androgens. Apart from the somatic cells, gonocytes represent a transient and finite phase of development leading to the formation of SSCs. Gonocyte development involves phases of quiescence, cell proliferation, migration, and differentiation [32]. Markers related to testicular cell development were selectively studied. The present study observed that a low dose of MEHP induced developmental disruption of fetal germ cells and testicular somatic cells, accompanied by decreased testosterone production. It was previously reported that MEHP exhibited antiandrogenic effects at the low dose of 1 *μ*mol/L, which was within the range of the concentrations found in the plasma from pregnant women or in neonate cord blood [33]. In this study, we found that the gene expression of Amh was significantly inhibited by MEHP exposure, which may lead to disturbed testis development and finally lead to improper differentiation of germ cells as well as other somatic cells. It was also found that exposure to MEHP in an organ culture system induced Sertoli cell vacuolation and decreased the production of anti-Müllerian hormone in a time- and dose-dependent manner [34]. Among all cell types in the rat testis, Sertoli cells located at the periphery of the seminiferous cords begin to differentiate from 13.5 dpc to 14.5 dpc. The development of the male reproductive system is firstly induced by AMH, which is produced by the pre-Sertoli cells of the early testis. The presence of AMH and testosterone would promote the Mullerian duct regression and the Wolffian duct differentiation into accessory glands [35]. Furthermore, Sertoli cells could direct the development of other cell types in the early testis, including fetal Leydig cells, peritubular myoid cells, and endothelial cells [35]. These cell types aid in seminiferous cord formation by surrounding Sertoli cells and germ cells [35]; improper Sertoli cell function may finally lead to testicular cell development and differentiation disorder.

We also found MEHP downregulated gene expression of Leydig cell markers (Hsd3*β*), accompanied by decreased testosterone production. Physically, testosterone production starts to arise around 15.5 dpc and reaches a peak at 18.5 dpc in the rat [36]. Past studies revealed that in utero exposure to phthalates induced inhibitory effects on the expression of genes encoding steroidogenic enzymes [37], in a time- and dose-dependent manner. It was reported that MEHP at the concentration of 10^−4^ mol/L reduced testosterone levels to the threshold of detection and disorganized the testis [34]. In accordance with our study, they also found that MEHP at 10^−6^ mol/L exhibited antiandrogenic effects. The antiandrogenic properties of MEHP may probably result from direct action or indirect action. Evidence supported that MEHP exert direct action by targeting the Leydig cells based on the fact that the deleterious effects of MEHP chronically preceded that on germ cells and on Sertoli cells [34], and MEHP induced the suppression of testosterone production in isolated Leydig cells and the mouse MA-10 cells [38]. The indirect action was based on the fact that fetal Leydig cells arise after the appearance of SRY-expressing Sertoli cells, and therefore, the appearance and differentiation of fetal Leydig cells are probably regulated by factors derived from Sertoli cells [39].

Germ cell stages between primordial germ cells (PGCs) and spermatogonial stem cells (SSCs) are often indiscriminately named gonocytes, representing a single developmental stage [28]. Gonocytes begin to divide from 13.5 dpc and enter quiescent phase until postnatal day 2~3 [40]. Further study revealed gene expression of germ cell markers (Pdgfr*β*, Sohlh1, and Hsp90) after MEHP exposure was downregulated compared with control. Among all markers, Hsp90 is expressed in prenatal gonocytes and exerts multiple roles, including the responses to toxicants, regulation of cellular events associated with development and differentiation of tissues [41]. Hsp90 might also represent a molecular bridge between the EDCs and PDGF pathways in gonocytes [42]. It was identified that prenatal exposure to several EDCs was associated with altered expression of the PDGFRs in neonatal testis, with the strongest effect of PDGFR*β* in gonocytes [42]. Previous studies also revealed that several proteins belonging to the PDGF cascade, such as Raf1 and ERK1/2, were shown to be sensitive to Hsp90 inhibitors and depend on Hsp90 for their activity [43]. Therefore, the decrease in Hsp90 expression could change the response of gonocytes to PDGF. As the precursors of SSCs, improper gonocyte development would detrimentally affect the life-long reservoir of germline stem cells and reproductivity in the adulthood [28]. Among all regulators, the role of Sohlh in germ cell differentiation was highlighted in researches showing that Sohlh2 knockout mice exhibited improper germ cell differentiation. Sohlh proteins were also identified to regulate Gfra1, Sox3, and Kit gene expression [44].

Redox control is one of the most important regulatory mechanisms in testicular physiology, and potent antioxidant system protects testicular cells against ROS damage [45]. Culty et al. [12] reported that fetal testosterone biosynthesis was suppressed via activation of peroxisome proliferator-activated receptors (PPARs) after phthalate exposure; in this process, imbalance in prooxidant/antioxidant ratio was induced and reactive oxygen species (ROS) production was elevated [12]. Our previous studies revealed that exposure to MEHP at doses higher than human exposure level induced the impairment of testicular antioxidative enzyme activities, and oxidative stress aggravated testicular injuries as the doses increased [6, 7]. Other studies also observed an increased apoptosis rate of spermatocytes, atrophy of testis, and high sperm DNA fragmentation index together with elevated ROS level [46, 47]. The present study observed that a low dose of MEHP downregulated antioxidative genes and induced testicular mitochondrial oxidative injury, while the combined exposure to MEHP and genistein exhibited minor testicular injuries. Furthermore, antioxidative enzyme activity also showed different alternations after MEHP single exposure and the combined exposure. Cotreatment with genistein seemed to attenuate MEHP-induced adverse effects, suggesting that genistein may act as scavengers of ROS via activating Nrf2 and downstream genes, which was consistent with the ultrastructural changes in the testis. It is widely accepted that genistein has both estrogenic [48] and antioxidative effects [49]. Our previous in vivo study also revealed that after gestational exposure, DEHP at a relatively low dose altered the redox marker expression at PND3, while those markers seemed to be alleviated when combined with genistein, suggesting the involvement of cellular stress in short-term DEHP effects and a protective effect of genistein [22]. It is reasonable to speculate that genistein may exert a protective role in its combination with MEHP at a low dose in isolate fetal testis, highlighting the importance of assessing impacts across a range of low doses and mixtures. Moreover, the mixture exposure may act not simply in adding or subtracting manners of individual components [25, 50] and assessing reproductive risk based on a single chemical effect might not faithfully represent the true outcome of mixture exposure. Future experiments will involve detailed analysis of cellular and molecular events contributing to acute effects in testis development including epigenetic aberrations that may exert long-term perturbations in gene expression.

## 5. Conclusions

In this study, we established the fetal testis organ culture system and applied it to EDC study. Our results revealed that a low dose of MEHP could disrupt gonocyte, Sertoli cell differentiation, and Leydig cell function in fetal testis, accompanied by testicular oxidative injuries. However, coadministration of genistein at a low dose could partially alleviate MEHP-induced fetal testicular cell damage and exert protection by an antioxidative effect. Cotreatment of genistein at a low dose may have a promising future on its curative role for attenuating other EDC-induced reproductive disorders during early life. Based on the results, it can be speculated that dietary intake of isoflavones may make the fetal testis less susceptible to phthalate-induced injury.

## Figures and Tables

**Table 1:** Primer sets used for quantitative real-time PCR.

Gene name	Accession no.	Forward primer	Reverse primer
Gapdh	NM_017008.4	5-TGGGTGTGAACCACGAGAA-3	5-GGCATGGACTGTGGTCATGA-3
Nrf2	NM_031789.2	5-ACGGTGGAGTTCAATGAC-3	5-TGTTGGCTGTGCTTTAGG-3
Pdgfr*α*	NM_012802.1	5-GCTACACGTTTGAGCTGTCAAC-3	5-ATGGTGGTCATCCACAAGC-3
Cyp11a1	NM_017286.2	5-CACGCACTTCCGGTACTTGG-3	5-CGGATATTTCCAGCTCTGCAATCCG-3
Tspo	NM_012515.1	5-CGCAATGGGAGCCTACTTTGTGCG-3	5-GCCAGGAGGGTTTCTGCAAG-3
Hsd3*β*	NM_001007719.3	5-GACCAGAAACCAAGGAGGAA-3	5-CTGGCACGCTCTCCTCAG-3
Amh	NM_012902.1	5-CGGGCTGTTTGGCTCTGATTCCCG-3	5-GTGGGTGGCAGCAGCACTAGG-3
Wt-1	NM_031534	5-CGGTCGTCTTCAGGTGGTCGGACCG-3	5-GCACCAAAGGAGACACACAGGT-3
Tspo	NM_012515.1	5-CGCAATGGGAGCCTACTTTGTGCG-3	5-GCCAGGAGGGTTTCTGCAAG-3
Pdgfr*β*	NM_031525.1	5-ATGGACATGAGCAAGGATGA-3	5-GTCCGCGTATTTGATGTGTC-3
Dazl	NM_001109414.1	5-TGAAGTTGATCCAGGAGCTG-3	5-CCACTGTCTGTATGCTTCGG-3
Sod1	NM_017050.1	5-AGAGAGGCATGTTGGAGACC-3	5-TAGTACGGCCAATGATGGAA-3
Sod2	NM_017051.2	5-GGCTTGGCTTCAATAAGGAG-3	5-TAGTAAGCGTGCTCCCACAC-3
Cat	NM_012520.1	5-TTCATCAGGGATGCCATGT-3	5-GGGTCCTTCAGGTGAGTTTG-3
Hsp90*α*	NM_175761.2	5-TTTCGTGCGTGCTCATTCT-3	5-AAGGCAAAGGTTTCGACCTC-3
Tgf*β*	NM_021578.2	5-CCGCAACAACGCAATCTATG-3	5-AAGCCCTGTATTCCGTCTCC-3
Gfr*α*1	NM_012959.1	5-GTACTTCGCGCTGCCACT-3	5-GCTTTCACACAGTCCAGACG-3
Sohlh2	NM_001034961.1	5-AGCCAGCTCCAGTTGTCTGT-3	5-GATGCTGGATGAGGCAGT-3

## Data Availability

Data are available upon request.
